# Induction of General Anesthesia and Mask Ventilation With a Full-Face Continuous Positive Airway Pressure Mask in a Patient With a Nose Deformity

**DOI:** 10.7759/cureus.9475

**Published:** 2020-07-30

**Authors:** Tais G. O Bertasi, Raphael A. O Bertasi, Shaun E Gruenbaum, Eduardo S Rodrigues

**Affiliations:** 1 Anesthesiology and Perioperative Medicine, Mayo Clinic, Jacksonville, USA

**Keywords:** face mask, difficult airway management, continous positive airway pressure, interests in difficult airway and regional anaesthesia, facial reconstruction, oro-facial problems, continuous positive airway pressure (cpap)

## Abstract

Mask ventilation (MV) is an essential component of airway management and can be lifesaving in situations where the placement of a secure airway device proves challenging. Effective MV requires a seal to be created between the mask and the face to maintain patency of the external airway structures and can be difficult in the setting of facial abnormalities or facial trauma. Here we describe a case in which a continuous positive airway pressure (CPAP) mask was used for anesthesia induction and MV in an 85-year-old man who underwent a plastic surgery reconstruction of the left nasal dorsum and ala following a Mohs surgery, which had prevented the use of conventional face mask. An effective seal was achieved, and anesthesia was successfully induced with the mask. We reviewed the literature and discussed alternative approaches for face mask use in the setting of facial abnormalities where the use of a conventional mask is unfeasible.

## Introduction

Effective mask ventilation (MV) is essential for safe airway management during general anesthesia (GA). MV is a fundamental airway management skill for providing oxygen to a conscious, obtunded or unconscious patient and can be lifesaving in situations where the placement of a secure airway device proves challenging. Even in the most experienced hands, however, MV can be difficult or impossible in some patients. The incidence of difficult MV has been reported in the literature to range from 1.06% to 5% in the general surgical population undergoing GA [1-3]. Because effective MV requires a seal to be created between the mask and the face to maintain patency of the external airway structures, patients with facial anatomical abnormalities or facial trauma can present unique challenges to MV. In these patients, therefore, consideration of alternative approaches for effective MV might be necessary.

Typically, difficult MV can result from any of the following conditions: inadequate mask seal, which can create a low-resistance alternative path in which oxygen flows; increased air-flow resistance between the external airway structures (i.e., nose and mouth) and the lungs; or decreased lung or chest wall compliance that results in increased distal airway pressures [4]. To facilitate MV for patients of different facial shapes and sizes, several commercial face masks are currently available. For patients with facial deformities, however, creating an effective seal with the use of conventional masks might be impossible. Currently, there is no consensus on the best approach to MV in these patients, since each case is unique and should be considered with the patient’s specific anatomical abnormality in mind.

Here, we describe a case of airway management in an anesthetized patient with a history of prior nose surgery, which had prevented the use of a conventional face mask for MV. Informed consent statement was obtained for this study. We subsequently reviewed the literature and discussed alternative approaches for face mask use in the setting of facial abnormalities where the use of a conventional mask is unfeasible.

## Case presentation

An 85-year-old man underwent a Mohs surgery with subsequent plastic surgery reconstruction of the left nasal dorsum and ala due to a primary infiltrative basal cell carcinoma located on the left nasal dorsum measuring 0.8 x 0.7 cm. He had an extensive history of skin cancer with a previous Mohs surgery on the right nasal ala four months prior. In addition to the skin cancer lesions, the patient had a history of hyperlipidemia and spinal stenosis. The preoperative anesthesia team performed a comprehensive airway examination, which demonstrated a Mallampati score of II. An interview of the patient revealed a STOP-Bang score of 3, suggesting that the patient was at relatively low risk for obstructive sleep apnea [5]. The surgeon requested that the patient be under GA for the duration of the entire procedure.

**Figure 1:**
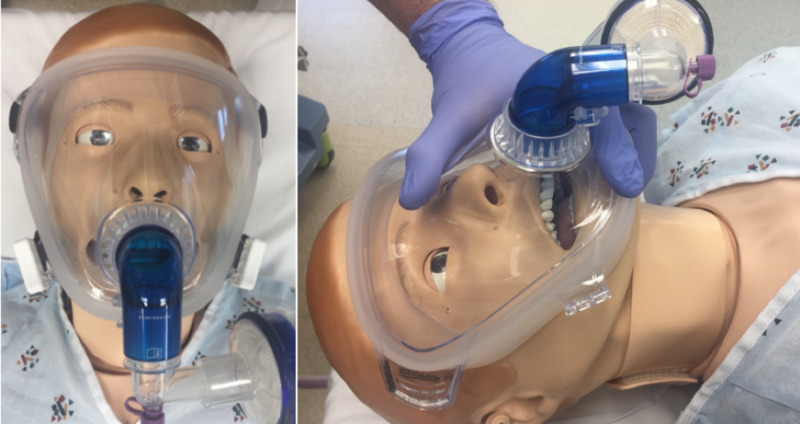
Full-Face CPAP Mask in a Manikin. Mask ventilation was achieved in our patient with the use of a full-face CPAP mask, as demonstrated here in this manikin. CPAP, continuous positive airway pressure.

Because the Mohs surgery was performed the day before the reconstruction, and because of the previous Mohs surgery on the right side, the use of a regular face mask was not feasible for MV. Therefore, a large continuous positive airway pressure (CPAP) mask (Respironics PerforMax Large, Philips Respironics, Murrysville, PA) was used for induction of GA and MV. Prior to induction of GA, the mask was secured to the face with the use of upper and lower straps that connect directly to the CPAP mask (Figure 1). A seal with the CPAP mask was confirmed by external examination of the face and mask, and by measurements of end-tidal carbon dioxide (CO_2_) and oxygen (O_2_) on the ventilator. After pre-oxygenation with a 1.0 fraction of inspired oxygen (FiO_2_) and end-tidal O_2_ > 90%, GA was induced with 4% sevoflurane in O_2_ via the CPAP mask. After the patient lost consciousness, positive pressure ventilation was performed through the CPAP mask with minimal effort. The patient was administered rocuronium 50 mg, and after three minutes of MV, the trachea was intubated on first attempt via video laryngoscopy with a size 7 endotracheal tube. Endotracheal intubation was confirmed by sustained measurement of ETCO_2_ on the ventilator. After completion of the procedure, the patient was extubated successfully, and the CPAP mask was re-applied to the patient’s face. The patient’s SpO_2_ concentrations were maintained ≥99% during the entire perioperative period.

## Discussion

Difficult MV in the unconscious patient is defined as the inability to provide ventilation due to gas leak, improper mask seal and/or resistance of gas ingress [6]. Identifying which patients might be at increased risk for difficult MV is essential for establishing a safe anesthetic plan prior to induction of GA. Several patient factors have been previously shown to be associated with a higher rate of difficult MV, including the presence of beard, obesity, macroglossia, age above 55 years, a history of snoring and abnormal facial anatomy [3,7]. Anesthesiologists must identify these factors prior to induction of GA, and be prepared to institute maneuvers that can improve the success of MV, including a two-handed MV technique, insertion of an oropharyngeal airway and by shaving the patient’s beard prior to surgery [8]. When the presence of a facial anomaly is predicted to impede adequate MV by interfering with a mask seal, other measures should be considered before proceeding with induction of GA.

Despite the commercial availability of several different types of masks designed for MV use, the use of these masks might not be feasible in cases where facial anomalies such as deformities, tumors, burns and injuries can impede the ability to create an adequate mask seal. Currently, there is a lack of dedicated studies that have focused on alternatives to MV in patients in whom a conventional facial mask is not feasible. Moreover, the use of an unfamiliar mask in a patient that is inherently at increased risk for failed MV carries its own challenges. This point is underscored by a prior study which reported a high variability in the performance (defined as a percentage of gas leak) and satisfaction among anesthesiologists when using different types of face masks to ventilate a manikin [9]. This suggests that the facial mask design in of itself can impact the adequacy of ventilation, regardless of any other underlying factors that are anticipated to make MV difficult.

For our patient, the anesthesia team recognized that the use of a conventional face mask would not be feasible since it would potentially damage the prior reconstructive surgery. With the use of a larger CPAP mask, in which the entire face was covered, an adequate seal and effective MV was achieved with the nose free from any external pressure. The adequate seal was further demonstrated by the successful induction of GA with sevoflurane that was delivered through the mask.

**Figure 2:**
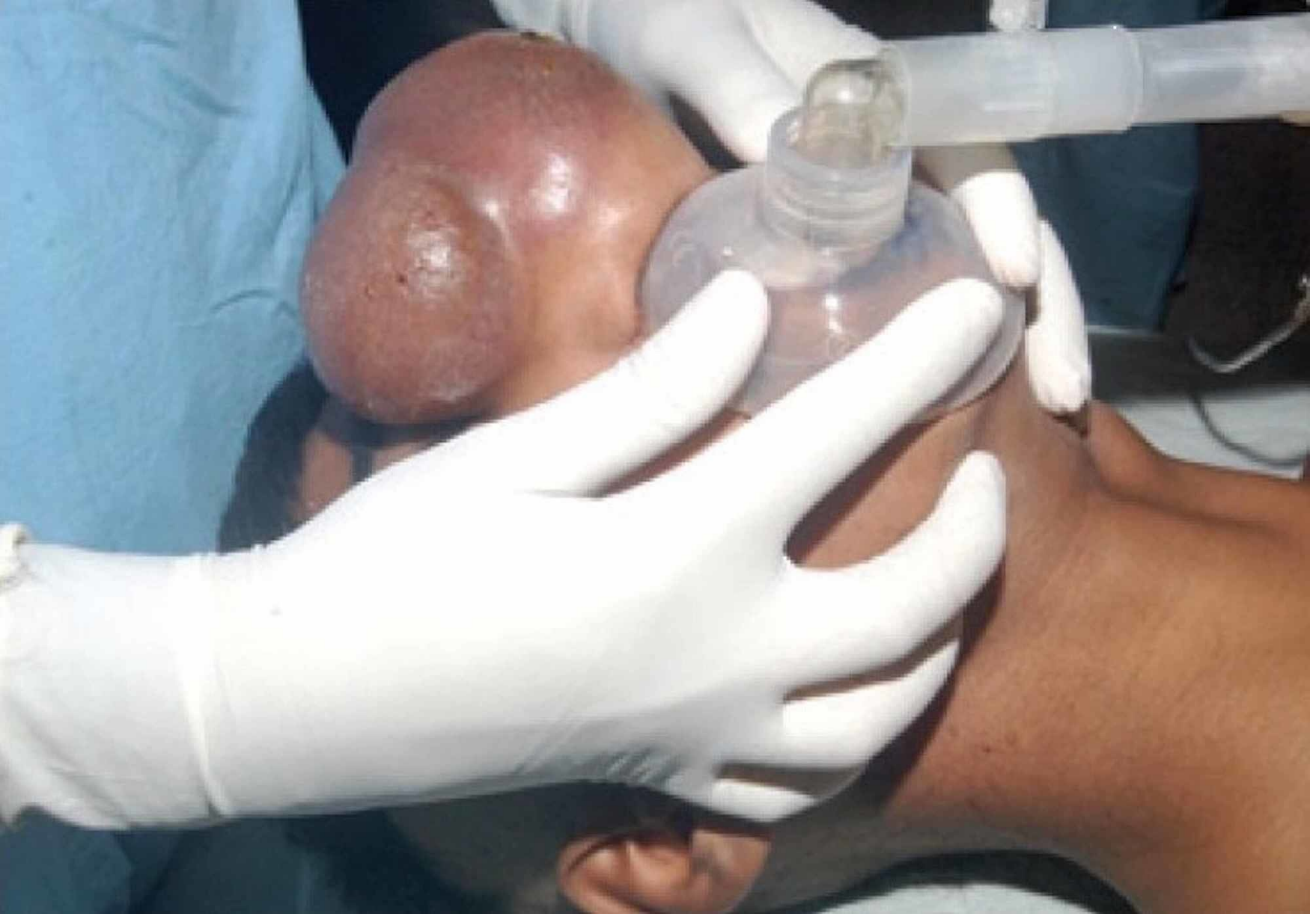
Pediatric Face Mask in an Adult With Nose Tumor. A pediatric-sized mask was used to ventilate the patient through the mouth only. Picture was adapted from Sethi et al., reproduction permission granted by Indian Journal of Anaesthesia [12].

Previous case reports have described other alternative approaches designed to overcome specific facial anomalies and aid in successful MV. Two papers described cases in which patients with large nose tumors prevented optimal seal of a facial mask. In these cases, a laryngeal mask airway was used as an alternative to oxygenate and ventilate the patient prior to endotracheal intubation [10,11]. Another case, in which a patient had a tumor that covered the entire nose, a pediatric-sized face mask (size number 2) was used to ventilate the adult patient through mouth only (Figure 2) [12].

**Figure 3:**
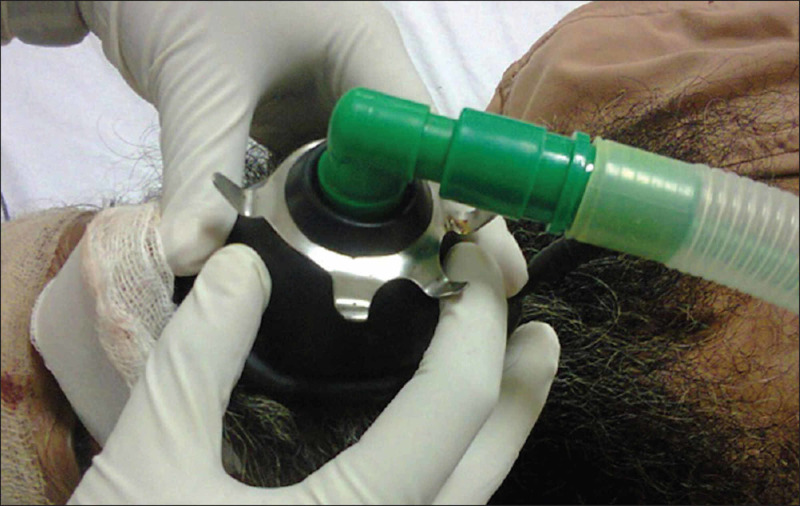
Conventional Face Mask in the Inverted Position. A conventional face mask applied in the inverted position was successfully used in a patient after a rhinoplasty procedure. Picture was adapted from Bajwa et al., reproduction permission granted by National Journal of Maxillofacial Surgery [14].

In another case, the use of a Rendell Baker Soucek mask was described for isolated ventilation through the nose in a patient with a massive neurofibroma that covered the entire right half of the patient’s face [13]. Lastly, a conventional face mask applied in the reversed position over the patient’s mouth has been previously described in a post-rhinoplasty patient (Figure 3) [14].

Inhalation agents may be preferred for GA induction when spontaneous breathing is desired. In the case of airway obstruction, there is a decreased uptake of the anesthetic and the patient is gradually re‐awakened, preventing the occurrence of apnea [15].

Sevoflurane contains low blood-gas solubility coefficient resulting in a rapid onset and offset, is sweet-smelling with low pungency and presents bronchodilation characteristics [16]. Compared to isoflurane and desflurane, it is the least irritating volatile; therefore, it is the agent of option for inhalational induction [17].

When total intravenous anesthesia is selected for GA induction, propofol is usually the drug of choice because of its rapid onset and smooth recovery profile, associated to its antiemetic properties. Another advantage is the short duration of action (5-10 minutes), which is beneficial in the case of a difficult airway management in which the patient needs to be awakened [18]. However, when comparing anesthesia induction with sevoflurane to propofol, a meta-analysis of 12 randomized controlled studies found higher incidence of apnea in the intravenous anesthesia group [19].

Thus, being able to perform anesthesia induction with inhalation agents when airway obstructions may exist is of great importance to maintain spontaneous breathing and avoid airway management complications.

To our knowledge, we described here for the first time the use of a CPAP mask for induction of GA and MV in a patient in whom a conventional face mask was not feasible. Although several alternative options for MV have been previously described for patients in whom a conventional mask was deemed unfeasible, only one of these approaches (via Rendell Baker Soucek mask) presented the possibility of induction of GA with volatile agents through the mask in patients with facial abnormalities [13].

## Conclusions

Studies that identify and compare alternatives approaches for MV in patients with facial abnormalities are currently lacking. This case describes the use of a CPAP mask to safely and effectively induce GA and ventilate a patient in whom the use of a conventional mask was deemed unfeasible. Because a CPAP covers the entire face without any external compression of the nose or mouth, it might offer some advantages for patients with facial abnormalities and should therefore be considered whenever appropriate.
